# Supplementary material for: Cytomegalovirus Glycoprotein B Genotype in Patients with Anterior Segment Infection
Source: Int J Mol Sci. 2023 Mar 27;24(7):6304. doi: 10.3390/ijms24076304 (PMC10094332; doi:10.3390/ijms24076304)
Supplement: Supplementary file 1 [file ijms-24-06304-s001.zip › ijms-2267171-supplementary.pdf]

**Supplementary Table S1**. Multiplex primers for cytomegalovirus glycoprotein B.

| Target | Sequence | Product (bp) |
|---|---|---|
| External F | TTTGGAGAAAACGCCGAC | 751 |
| External R | GCGGCAATCGGTTTGTTGTA | |
| Inner F1 | ATGACCGCCACTTTCTTATC | 420 |
| Inner F2 | TTCCGACTTTGGAAGACCCAA | 613 |
| Inner F3 | TAGCTCCGGTGTGAACTCC | 190 |
| Inner F4 | ACCATTCGTTCCGAAGCCGAGGAA | 465 |
| Inner F5 | TACCCTATCGCTGGAGAAC | 139 |
| Inner R | GTTGATCCACACACCAGGC | |
